# Early prevention of anxiety disorders in young children: The implementation of a live, online, targeted group-based parenting program^☆^

**DOI:** 10.1016/j.invent.2026.100956

**Published:** 2026-05-25

**Authors:** Nina L. Komrij, Mia P. Kösters, Frederike Y. Scheper, Megan F.S. Soppe, Leonie J. Vreeke

**Affiliations:** a Leiden University, department of Developmental and Educational Psychology, Institute of Psychology, Leiden, the Netherlands; b Public Health Service Amsterdam, department of Healthy Living, Amsterdam, the Netherlands; c MOC ‘t Kabouterhuis, department of Infant Mental Health, Amsterdam, the Netherlands

**Keywords:** Mental health, Anxiety disorders, Prevention, Young children, Parenting, Cool Little Kids, Process evaluation

## Abstract

Anxiety problems are highly prevalent and often develop during early childhood. Targeted, group-based parenting programs have shown promise in preventing anxiety disorders in young children. However, these programs can be challenging to implement and comprehensive evaluations of their implementation in real-world settings remain scarce. Therefore, this study investigated the implementation of an online group-based anxiety prevention program for parents of anxiety-prone toddlers in a naturalistic setting in the Netherlands. A hybrid effectiveness-implementation design was used, including participants involved in a randomized controlled trial (*n* = 156) and those who received the intervention outside the trial (*n* = 45), representing parents of 201 children in total. Recruitment and delivery were facilitated through collaboration with child professionals, Public Health Services, and mental health organizations. Implementation indicators included recruitment, reach, dose delivered and received, fidelity, satisfaction, sustainability, and context. These were assessed using parental questionnaires, intervention observations, qualitative interviews, and research logs. The intervention was largely implemented as intended with adequate recruitment, high fidelity and satisfaction, and sustained use of the intervention strategies over time. However, the population reached predominantly consisted of highly educated parents with a non-migrant background, limiting the generalizability of the findings to more diverse populations. This study demonstrated the potential of implementing an online, group-based parenting anxiety prevention program to increase accessibility while maintaining high-quality delivery and participant satisfaction. Addressing challenges related to inclusivity could enhance the intervention's reach and impact, enabling it to play a significant role in early prevention of anxiety disorders across diverse populations and settings.

## Highlights

• Implementing a live, online group-based anxiety-prevention program is feasible.
• Online attendance was higher than in prior in-person anxiety prevention programs.
• Parents valued the flexibility and accessibility of the intervention.
• Tailored implementation strategies are needed to reach more diverse populations.

## Graphical abstract

**Figure:**
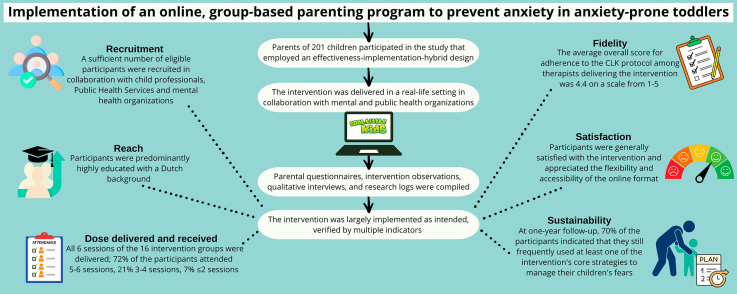

## 1 Introduction

Anxiety disorders represent a significant public health concern due to their high prevalence, early onset in life and detrimental impact on both well-being and overall health (Rapee et al., 2023; Polanczyk et al., 2015). Despite their prevalence, anxiety problems in children remain frequently undetected and untreated, resulting in profound and enduring consequences on children's development, alongside significant societal costs (Pollard et al., 2023; Kazdin, 2017). This underscores the need for early preventive interventions.

One risk factor that offers opportunities for early prevention of anxiety disorders is behavioral inhibition (BI), a heritable, temperamental trait characterized by a consistent tendency to display fear and withdrawal in unfamiliar situations (Fox et al., 2005). BI is already detectable in toddlerhood and remains moderately stable over time (Chronis-Tuscano et al., 2009). Research consistently demonstrated that BI is a key risk factor for later anxiety disorders. According to a recent meta-analysis, early childhood BI is linked to nearly three times higher odds of developing anxiety symptoms and almost six times higher odds of developing social anxiety disorder (Sandstrom et al., 2020). Furthermore, parents' responses and attitudes toward their child's inhibited behaviors can either mitigate or exacerbate the development of anxiety disorders. For example, controlling parenting styles may increase this risk by limiting opportunities for the child to develop the skills needed to cope with potential anxiety-provoking situations, whereas parental warmth may serve as a protective factor (Yap and Jorm, 2015; Warner and Strawn, 2023). Thus, given parents' critical role in children's early development and the early onset of anxiety disorders, targeting parents of preschool-aged children with elevated BI has the potential to achieve significant preventive impact.

The Australian Cool Little Kids (CLK) intervention, a group-based parenting program, illustrates this preventive approach by educating parents of inhibited children about anxiety, associated risk factors, the influence of their own behaviors, and techniques to manage their child's fears (Rapee et al., 2005). Research on the CLK intervention in Australia has demonstrated good efficacy, with preventive effects persisting up to eleven years post-intervention (Rapee, 2013). However, a subsequent translational trial revealed no sustained effects on internalizing problems at the three-year follow-up assessment (Bayer et al., 2022). The authors suggest that the differences in effectiveness may partly be due to lower engagement of parents in real-world settings (Bayer et al., 2022) compared to a controlled research setting (Rapee, 2013). Similar differences in intervention effectiveness between controlled research settings and naturalistic settings have been observed in other studies investigating (preventive) interventions (Dane and Schneider, 1998; Marchand et al., 2011), underlining the need to move beyond outcome evaluation alone and consider the contextual factors that influence how interventions unfold in practice (Moore et al., 2015).

These differences in effectiveness may be attributed to implementation-related factors, including fidelity, participant satisfaction, and contextual conditions. Nonetheless, such factors are often not examined in traditional intervention studies (Williams and Beidas, 2019). Investigating these implementation aspects is particularly relevant for group-based interventions, which often face significant barriers when embedded in routine care, such as travel time and transportation challenges, despite promising evidence of their effectiveness (Olofsson et al., 2016; Cooper et al., 2022). To address these barriers and support more effective implementation, innovative online delivery formats represent a promising alternative. Online interventions can reduce these common logistical obstacles and are often preferred by parents from diverse backgrounds (Duppong-Hurley et al., 2016; Broomfield et al., 2021; Flujas-Contreras et al., 2019). When delivered in a live, interactive online format, the key benefits of face-to-face delivery, such as real-time interaction and peer support, are preserved, potentially enhancing the real-world implementation of parenting programs.

Despite the promising developments to improve implementation and the increasing recognition of the importance of implementation research over the past decade (McHugh and Barlow, 2010; Proctor et al., 2023), research on psychological interventions still predominantly focuses primarily on investigating intervention efficacy (Williams and Beidas, 2019). To our knowledge, very few studies have comprehensively examined the real-world implementation of childhood anxiety prevention programs, with most existing studies relying on limited implementation-related measures, such as participant satisfaction or acceptability or small-scale feasibility studies (e.g., Tozzi et al., 2018; Zhou et al., 2019). As a result, essential factors that contribute to successful implementation, such as recruitment strategies, fidelity, and long-term sustainability are often overlooked. To truly impact public health, the implementation of (preventive) interventions should be examined as rigorously as their efficacy.

Therefore, this study aimed to comprehensively investigate the implementation of an online, targeted, group-based parenting anxiety-prevention program in the Netherlands. The findings will provide essential insights to inform future implementation strategies and enhance the impact and long-term success of group-based preventive interventions (Moore et al., 2015; Proctor et al., 2023; Durlak and DuPre, 2008).

## 2 Methods

### 2.1 Design

The current study was guided by recommendations for process evaluations of the UK Medical Research Council (Moore et al., 2015) and part of a larger research project referred to as ‘the Cool Little Kids project’ that aimed to investigate the implementation and (cost-) effectiveness of an adapted version of the CLK intervention in the Netherlands. Therefore, an effectiveness-implementation hybrid design was employed (Landes et al., 2020), utilizing a randomized controlled trial (RCT) comparing the adapted CLK intervention to an active control condition (a book with general parenting advice), to investigate both effect and implementation outcomes. The trial was prospectively registered in the Netherlands Trial Register (NL9633) on July 27, 2021 and was approved by the Medical Ethics Committee Leiden The Hague Delft (N21.079; not subject to the Medical Research Involving Human Subjects Act) and the Psychology Research Ethics Committee of Leiden University (V2–3346). The current evaluation only focused on the implementation outcomes, the results on the effectiveness of the intervention are described elsewhere (Komrij et al., under review).

### 2.2 The intervention

**Fig. 1:**
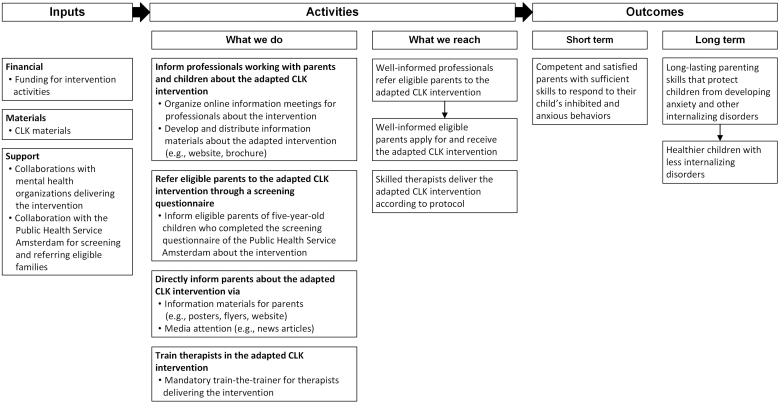
Logic model for the implementation of the Dutch adapted CLK intervention.

The intervention was a Dutch adaptation of the Australian CLK intervention, a targeted group-based parenting program aiming to prevent the development of anxiety disorders in anxiety-prone toddlers with elevated BI (Rapee et al., 2005). The main goals of the intervention are to teach parents (i) about (the risk factors for) anxiety disorders in young children, (ii) about the impact of overprotective parenting behaviors on their child's anxiety, (iii) how to support and help their child in managing their fears, and (iv) how to deal with their own worries. These goals are translated into three core strategies that parents are taught and encouraged to apply in their daily lives: (i) adopting non-overprotective approaches to cope with the child's inhibited behaviors, (ii) implementing in-vivo exposure through anxiety hierarchies, and (iii) practicing realistic thinking to address parents' own worries. The original CLK intervention comprised six, manualized 90-min sessions in groups of parents of six to eight children, delivered over approximately ten weeks. The training uses cognitive behavioral therapy (CBT), including psychoeducation, in-vivo-exposure techniques, and cognitive restructuring, supported by a parental workbook with homework assignments for each session. In the present study, the CLK intervention was delivered in accordance with the Dutch translation of the original protocol, but adapted to a live, online format using a videoconferencing platform to ensure continuity during the COVID-19 pandemic and to enhance accessibility for parents residing in geographically remote areas. The intervention was guided by two therapists per group who were working for a mental health organization within the Netherlands. The therapists were required to (i) have an (applied) university degree in Psychology or Child Studies, (ii) have training in applying CBT and standard therapeutic skills, and (iii) complete the Dutch CLK train-the-trainer program. Fig. 1 illustrates the logic model of the intervention, providing an overview of the planned activities, their outputs the theorized relationships between these activities, and the short- and long-term outcomes of the intervention.

### 2.3 Participants and procedures

Participants were the parents of 201 children who participated in the CLK project, divided into three distinct sets. Set A consisted of three groups of parents who were all allocated to the CLK intervention (*n* = 21). Set B consisted of ten groups of parents who participated in the RCT and were randomly allocated to either the CLK intervention (*n* = 78) or the active control condition (n = 78). Set C consisted of another three groups of parents who were included after recruitment for the RCT ended and were all allocated to the CLK intervention (*n* = 21). Table 1 provides an overview of the data collection methods used for parents from sets A, B, and C. Additionally, a visual flowchart can be found in Appendix 1.

**Table 1:** Overview of the methods used and implementation indicators.

	Pre-intervention questionnaire	Post-intervention questionnaires	CLK observations	Qualitative interviews	Research logs
Recruitment	ABiBcC				R
Reach	ABiBcC				
Dose delivered					R
Dose received		ABiC	ABi		
Fidelity			ABi		
Satisfaction		ABiC		C	
Sustainability		ABi			
Context					R

*Note**.*** A = Set A - measured among parents who were all assigned to the CLK intervention as randomization was not implemented due to recruitment challenges in the early phase of the study.

Bi = Set B intervention group - measured among parents participating in the intervention group of the RCT.

Bc = Set B control group - measured among parents participating in the control group of the RCT.

C = Set C - measured among parents who were included after the inclusion for the RCT ended and were all assigned to the CLK intervention.

R = measured through general research logs.

To be included in the study, participants were required to (i) demonstrate adequate proficiency in the Dutch language and (ii) provide written informed consent. Additionally, their child had to (iii) be between the ages of two and six years, and (iv) exhibit a high level of BI, as indicated by a score greater than 42 on the Behavioral Inhibition Questionnaire - Short Form (BIQ-SF). This cut-off was based on the 80th percentile of BIQ-SF scores from a community sample (Vreeke et al., 2012), in line with evidence that elevated levels of BI are observed in roughly 20% of children (Chronis-Tuscano et al., 2009). Participants were excluded if (v) they were undergoing acute psychiatric treatment, (vi) their child was receiving treatment for an anxiety or another mental disorder, or (vii) their child's anxiety symptoms already caused significant interference with daily functioning, such that a referral to a treatment program was considered more appropriate than participation in the present preventive intervention. The criteria were checked using a registration form and through a brief structured telephone intake interview with a trained research assistant. In case of doubt, the wider research team was consulted. Following confirmation of eligibility, one parent per child provided informed consent prior to formal inclusion in the study. This parent was invited to participate in the research assessments, while both parents were invited and encouraged to participate in the intervention.

In August 2022, before including the fourth group of participants in the RCT (set B), we decided in consultation with the research team and the Australian developer of the original CLK intervention to change the allowed age range (criteria iii) of the children from two to six to three to six years. This decision was based on feedback from the therapists, who indicated that the intervention techniques appeared less suitable and that the children's behavior was often complex and difficult to define due to their young age. Parents of two-year-old children who had already been enrolled (*n* = 7) were retained for the purposes of the current study.

Existing public health promotion and prevention recruitment strategies were used to reach eligible parents, see also Fig. 1. Parents were recruited in collaboration with the Public Health Services and the mental health organizations that were involved in delivering the intervention. First, parents were recruited by using a screening questionnaire called ‘Jij en Je Gezondheid’ (translation: ‘You and Your Health’; JEJG) that is used by the Public Health Service Amsterdam and three other provincial Public Health Services in the Netherlands (Public Health Service Amsterdam, 2025). Every parent living in one of these areas with a child aged five years is invited to fill in this questionnaire preceding the regular health check with a youth health care professional. The BIQ-SF (Vreeke et al., 2012), measuring the levels of BI in the child, is incorporated in this questionnaire. Parents who scored above the BIQ-SF cut-off score were informed about the possibility of participating in the CLK project. Second, we informed youth healthcare professionals about the research project through presentations during their regular meetings and by distributing posters, flyers, and a brochure with information about the project. We asked these professionals to refer any potentially eligible parents to the project. Third, we targeted parents directly by distributing posters and flyers at relevant public places (e.g., schools, daycare centers, libraries). We also approached local and national media outlets, resulting in several online news articles about the project.

### 2.4 Implementation indicators

To investigate the implementation of the intervention, we assessed several implementation indicators: *recruitment, reach, dose delivered and dose received, fidelity, satisfaction, sustainability,* and *context* (Proctor et al., 2023; Saunders et al., 2005). *Recruitment* was defined as the strategies and procedures that were used to recruit parents for the CLK intervention. *Reach* was referred to as the number and characteristics of parents who expressed interest and actually participated in the CLK project. *Dose delivered* was the number of intervention groups and sessions that were delivered by the therapists and *dose received* referred to the extent to which parents were exposed to and engaged with the intervention content (Saunders et al., 2005). *Fidelity* was defined as the extent to which the therapists delivered the intervention conform to the CLK protocol, including the assessment of general therapist skills considered important for facilitating group-based interventions (Gladding, 2003). *Satisfaction* was determined by the opinions of the participants about the different aspects of the intervention. *Context* was referred to as factors that hindered or facilitated the implementation of the online intervention. Lastly, *sustainability* was described as the intentions and actual continued use of the strategies and techniques learned by parents during the intervention in the longer term.

### 2.5 Measures

To assess the different implementation indicators, we employed multiple methods (i.e., parental questionnaires, qualitative interviews, observational data, and research logs). The different methods complemented each other, ensuring a comprehensive evaluation. See also Table 1 and Appendix 1.

#### 2.5.1 Parental questionnaires

All participating parents (one parent per child) were asked to fill in a questionnaire at baseline and post-intervention. Additionally, parents from sets A and B were also invited to fill in a questionnaire at six- and twelve-month follow-up. All the questionnaires were compiled specifically for this research project, inspired by previous research (Garcia-Smith and Effken, 2013; Koldijk, 2019).

To assess *recruitment* and *reach*, we gathered information about the means by which the participant had heard from the CLK project, and their sociodemographic characteristics (i.e., age, sex, country of birth, parental educational level, household income, family composition, and city of residence). We also included one post-intervention question related to *dose received*, asking whether participants had discussed the content of the sessions with their partner. This was intended as an additional and indirect indicator of parents' exposure to the intervention content, particularly in cases where only one parent attended the sessions. To assess *satisfaction*, we asked participants to which extent they were satisfied with the CLK intervention in general, its content, its environment, and their self-assessed attainment of the four intervention goals. Participants could answer on a five-point Likert scale ranging from (1) ‘completely disagree’ to (5) ‘completely agree’. At the end of the questionnaire, participants also had the opportunity to leave any additional comments about their experiences with the intervention in an optional open-ended question, which were incorporated into the qualitative analyses (see section 2.6). To assess *sustainability*, the six- and twelve-month follow-up questionnaire asked if participants were still implementing the three core strategies of the intervention in their daily lives (see section 2.2). Participants could answer on a five-point Likert scale ranging from (1) ‘(almost) never’ to (5) ‘(almost) always’. Due to low variability, the answers were categorized into a three-point scale: ‘rarely’ (i.e., ‘(almost) never’ and ‘rarely’), ‘sometimes’, and ‘frequently’ (i.e., ‘often’, and ‘(almost) always’. Participants who indicated that they rarely implemented a strategy were asked to provide their reasons.

#### 2.5.2 Intervention observations

All sessions of the thirteen intervention groups that were delivered to parents from sets A and B were observed by NK and twelve trained Psychology master's students, using predefined observation forms. The observation forms were designed specifically for the current study and inspired by the outline of previous studies (Koldijk, 2019; Kösters et al., 2017). The first part assessed the extent to which the therapists adhered to the protocol. These observation items were based on the aims of the intervention and its related activities described in the protocol for each session, thus measuring *fidelity.* The second part assessed general skills of the two therapists (e.g., the extent to which the therapists stimulated interaction between the parents and showed empathy), also belonging to *fidelity* (Gladding, 2003)*.* The third part assessed the attendance and active participation of the parents (e.g., the extent to which the parent was actively participating in the session), belonging to *dose received*. The second and third part of the observation form were identical for all sessions.

All items were scored on a four-point Likert scale ranging from (1) ‘not at all’ to (4) ‘very good’. The first session of each intervention group was jointly observed by NK and the student observer to align their interpretation of the scoring criteria and minimize discrepancies, after which the subsequent sessions were observed by one observer. To enhance the consistency of the observation scores and support reliable scoring across observers and sessions, NK developed a scoring protocol, outlining the criteria for each response option. In addition, observers were required to provide a written justification for each score based on their observations. These justifications were reviewed by NK to check whether the assigned scores were consistent with the scoring protocol and supported by the observations. If discrepancies were identified, scores were discussed with the observer and adjusted if necessary, although this was rarely required.

#### 2.5.3 Qualitative interviews

To gain a deeper understanding of participants' *satisfaction* with the adapted CLK intervention, individual, semi-structured qualitative interviews were conducted in February and March 2024 with participants from one intervention group within set C. All parents who participated in at least one of the sessions of this intervention group (*n* = 8) were invited to participate. A purposive sampling strategy was employed to select this specific group, as parents in other groups were already involved in additional measures and interviews for the larger CLK research project, which would have significantly increased their workload. This resulted in six participants: two fathers and four mothers of six children in total.

Interviews were conducted via an online videoconferencing program by NK and MS. NK had prior training and experience in conducting and analyzing qualitative interviews, while MS was trained by NK and had prior experience with conducting online interviews. To ensure methodological soundness, qualitative methods experts were consulted throughout the data collection and analysis process. Interviews lasted 26 to 46 min and were guided by a topic list that was based on the satisfaction questionnaire. Therefore, topics included participants' experiences with the content and environment of the intervention. At the end of each interview, the interviewer summarized the participant's response to ensure accurate interpretation, allowing participants to clarify or expand their answers if necessary. All interviews were recorded and transcribed verbatim wherein all identifying information was removed. Participants did not receive their transcripts.

The transcripts were analyzed using thematic analysis within a framework approach. Interviews were coded using a combination of deductive and inductive coding in Atlas.ti version 9. An a priori codebook was developed from the satisfaction questionnaire that guided the initial deductive coding. When the data did not fit within the pre-established codes, additional codes were developed inductively. All inductive codes fitted within the main deductive themes and no new themes emerged. To systematically structure the findings, codes were categorized under predefined labels ‘satisfied experience’, ‘neutral experience’, ‘dissatisfied experience’, and ‘suggestion for improvement’. Coding was completed independently by NK and MS to ensure reliability. Any disagreements in coding were discussed and resolved collaboratively.

#### 2.5.4 Research logs

Throughout all phases of the research project, the CLK research team maintained detailed research logs, comprising anonymized notes on all relevant aspects of implementation, including communication with the participating mental health organizations and therapists about the planning and execution of the CLK intervention groups and (potentially interested) participants. In addition, the number of participants who expressed interest in participating in the CLK intervention was also included in the logs. Therefore, these data were used for assessing *recruitment*, *reach*, *dose delivered*, and *context.*

### 2.6 Data analysis

Quantitative data were analyzed using IBM SPSS Statistics version 29. Descriptive statistics were calculated for the questionnaire and observation data to assess *recruitment* and *reach* (i.e., means by which participants had heard from the CLK project and demographics)*, dose received* (i.e., parents' attendance, active participation, and interaction)*, fidelity* (i.e., overall score for adhering to the CLK protocol and therapists' skills scores)*, satisfaction,* and *sustainability* (i.e., long-term enactment of the intervention strategies). To further explore *fidelity*, a Friedman's test was conducted to determine whether the adherence scores significantly differed across sessions (α = 0.05; two-sided). A non-parametric test was chosen due to the small sample size of the thirteen groups, which violates the assumptions of parametric tests.

To gain deeper insights into participants' *satisfaction*, a sequential explanatory mixed-methods design was employed. The qualitative data, comprising the interviews transcripts and responses to the open-ended questionnaire item regarding parents' satisfaction, were used to provide deeper insights and contextualize the quantitative findings. This approach enabled the integration of both data sources, facilitating a more comprehensive understanding of participants' *satisfaction.* Qualitative data were reported according to the Standards for Reporting Qualitative Research (SRQR) checklist (O'Brien et al., 2014).

## 3 Results

### 3.1 Recruitment and reach

Between July 2021 and May 2024, parents of a total of 506 children expressed interest in participating in the intervention. Parents of a total of 201 children (40%) met inclusion criteria and participated in the CLK project (see Appendix 1 for the participant flowchart). The primary reasons for non-participation included limited Dutch proficiency, referral to specialized mental health care based on the telephone intake, diminished interest after receiving project information, or a loss of contact.

Among the participating parents, 21.5% indicated that they were referred by a professional (e.g., youth health care worker, primary school teacher), 16.4% applied after filling in the JEJG questionnaire, 35.6% applied after reading one of the news articles about the research project, 7.3% were referred by an acquaintance, 11.9% found the CLK project via another way (e.g., via posters, flyers or the internet), and 7.3% did not specify how they found the CLK project. In Table 2, an overview of the participant characteristics is presented.

**Table 2:** Sociodemographic characteristics of the parents participating in the CLK project (*n* = 199).

Parents' characteristics	
Age, M (SD)	37.4 (4.5)
Questionnaire filled in by the child's mother, % (n)	85.9 (171)
Parental role of the parent's partner, % (n)	
*No partner or partner is not a parent of the child*	8.0 (15)
*Partner is the child's other (biological) parent*	85.5 (172)
*Unknown*	7.0 (14)
Country of birth, % (n)	
*Both parents born in the Netherlands*	72.6 (146)
*One of the parents not born in the Netherlands*	22.4 (45)
*Both parents not born in the Netherlands*	5.0 (10)
Parental educational level a, n (%)	
*Low*	1.5 (3)
*Medium*	7.7 (15)
*High*	90.8 (178)

Children's characteristics	
Age, M (SD)	4.6 (1.1)
Girls, % (n)	58.2 (117)
Born in the Netherlands, % (n)	94.5 (190)

Family characteristics	
Gross monthly household income, % (n)	
*0 to 3000 euros*	6.0 (12)
*3000 to 6000 euros*	26.4 (53)
*6000 to 9000 euros*	28.8 (58)
*More than 9000 euros*	20.9 (42)
*Unknown*	17.9 (36)
Other children in the household, % (n)	
*No*	18.9 (38)
*Yes, one sibling*	69.7 (140)
*Yes, more than one sibling*	10.6 (21)

*Note* For each child, one parent was asked to fill in the questionnaires. Parents of two children did not complete the baseline questionnaire, therefore the total number of respondents at baseline was 199 instead of 201.

a Based on the highest completed educational level of both of the child's parents (Statistics Netherlands, 2025): low = primary education, prevocational secondary education, the first three years of upper secondary education, and lower vocational training; medium = upper secondary education from the fourth year onwards and intermediate vocational training; high = university or university of applied sciences.

### 3.2 Dose delivered and dose received

For the sixteen planned intervention groups, all six sessions were delivered as intended. Table 3 provides an overview of parents' attendance, active participation, and mutual interaction per session across the thirteen groups that were observed from set A and B (*n* = 99). 72% of these participants attended five or six sessions (either alone or with their partner), 21% three or four sessions, and 7% two or fewer sessions. Participants who attended the sessions generally actively participated and interacted with other parents, with average scores ranging from 3.51 to 3.75 and 2.75 to 3.82, respectively, on a scale from 1 to 4. At post-intervention, 67.5% of the parents indicated that they discussed the content of five or six of the sessions with their (ex-)partner, whether the partner was present or not. 19.5% of the participants discussed the content of three or four of the sessions, 11.7% the information of one or two of the sessions and only 1.3% of the participants indicated that they did not discuss any content of the intervention with their (ex-)partner.

**Table 3:** Parents' attendance and active participation during the sessions of the adapted CLK intervention (*n* = 99).

	No parent present, % (n)	One parent present, % (n)	Two parents present, % (n)	Active participation score for present parents, M (SD)	Interaction between parents, M (SD)
Session 1	3 (3)	52 (51)	45 (45)	3.51 (0.65)	2.75 (0.90)
Session 2	11 (11)	47 (46)	42 (42)	3.70 (0.55)	3.29 (0.84)
Session 3	18 (18)	43 (42)	39 (39)	3.64 (0.47)	3.16 (0.87)
Session 4	17 (17)	46 (45)	37 (37)	3.60 (0.53)	3.08 (0.72)
Session 5^a^	30 (27)	46 (42)	24 (22)	3.75 (0.41)	3.82 (0.38)
Session 6	30 (30)	43 (42)	27 (27)	3.73 (0.44)	3.43 (0.76)

*Note.* Scores were calculated for the thirteen groups of parents from sets A and B and ranged from 1 (not at all) to 4 (very good).

a Observation data for the fifth session of one of the groups were lost, therefore the scores for the fifth session are based on data from twelve groups (*n* = 91) instead of thirteen.

Nine participants (9.1%) actively decided to stop following the intervention before the final session. Reasons included that their child's anxiety symptoms were too severe, prompting the parent to seek more intensive support (*n* = 2); limited time to participate in the intervention (*n* = 4); disagreements with the intervention techniques (*n* = 2); and discomfort with the group-format (*n* = 1).

### 3.3 Fidelity

In Table 4, a full overview of the therapists' skills scores is presented. The overall skills of the sixteen therapists during the six sessions ranged from 3.34 to 3.86 on a scale from 1 to 4. Furthermore, the average score for adherence to the CLK protocol was 3.48 (*SD* = 0.19; scale 1–4). The Friedman test indicated no significant differences in adherence scores across the six sessions (scores per session ranged from 3.36 to 3.63).

**Table 4:** Therapists' skills during the six sessions of the adapted CLK intervention.

	Weighted average (SD)
The therapists actively engaged all participating parents	3.79 (0.18)
The therapists encouraged interaction between the participating parents	3.34 (0.51)
The therapists made the participating parents feel comfortable	3.81 (0.21)
The therapists showed empathy toward the participating parents	3.86 (0.16)
The therapists responded adequately to parents' reactions and questions	3.78 (0.23)
The atmosphere during the sessions was pleasant	3.80 (0.27)

*Note.* Scores were calculated for each of the thirteen groups from sets A and B and weighted based on the number of participants in each group. Scores ranged from 1 (not at all) to 4 (very good).

### 3.4 Satisfaction

**Fig. 2:**
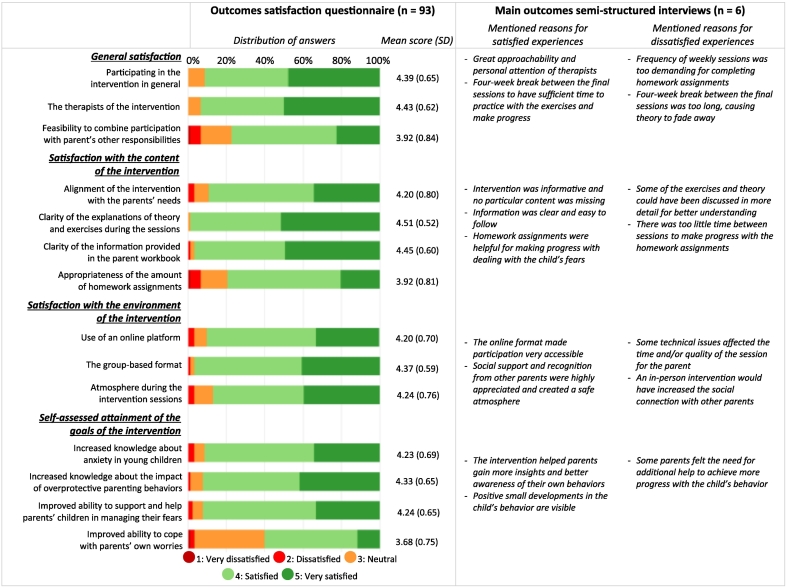
Parents' satisfaction with the adapted CLK intervention at post-intervention.

Fig. 2 presents the results of the satisfaction questionnaire, alongside the main findings from the semi-structured interviews. Appendix 2 provides interview quotations supporting the reported qualitative findings. In addition to the quantitative data from the questionnaire, 55 participants provided comments in response to the optional open-ended question about their experiences with the intervention, which were incorporated into the qualitative analyses.

#### 3.4.1 General satisfaction

Overall, parents reported high satisfaction, as reflected in both the questionnaire and interviews. At six- and twelve-month follow-up, satisfaction remained high with mean general satisfaction scores of 4.14 (*SD* = 0.72) and 4.13 (*SD* = 0.72), respectively (range 1–5). Participants were particularly satisfied with the therapists and appreciated their guidance and personal attention. Most participants also expressed their satisfaction with the feasibility of combining participating in the intervention with their other responsibilities. However, this question received more neutral responses compared to the other questions. The interviews reflected mixed experiences: some participants found the weekly sessions too demanding, as they lacked sufficient time to complete the homework assignments. They especially appreciated the four-week break between the final two sessions, as this gave them the time to practice implementing the intervention strategies in daily life. Other participants, however, mentioned that this break was too long, disrupting their routine and making it difficult to retain or apply the strategies.

#### 3.4.2 Satisfaction with the content of the intervention

Participants described the therapists' explanations and materials as clear, engaging and directly relevant to their situation. Several participants referred to the strategies as ‘eye-openers’. Moreover, participants emphasized the usefulness of completing the homework assignments to directly support their children. Critical notes included that some participants felt they could have derived greater benefit from the intervention if more time had been devoted to discussing certain exercises and theoretical aspects in addition to listening to other parents.

#### 3.4.3 Satisfaction with the environment of the intervention

The group-based format was widely praised for fostering social support and recognition, shared understanding, and practical tips from other parents. The online format was also well received, with many participants appreciating the accessibility it offered. The format made it easier for the participants and their partners to attend all sessions. Nonetheless, some participants expressed a preference for in-person sessions, believing these would foster stronger connections among participants, although they acknowledged that attending such sessions might have been logistically challenging. Additionally, some participants noted that technical issues occasionally disrupted the sessions, particularly when therapists were unfamiliar with resolving such problems.

#### 3.4.4 Satisfaction with the self-assessed attainment of the goals of the intervention

Participants were generally satisfied with their progress toward the intervention goals. The fourth goal (i.e., improved ability to cope with parents' own worries) received slightly lower scores compared to the other goals. This discrepancy could not be fully explained by the interview data. Some participants mentioned positive developments in their child's anxious behavior following the intervention. However, they particularly noticed changes in their own perceptions and behavioral responses to their child's fears. Additionally, while some participants felt that they still needed more support for their child, they expressed that the CLK intervention helped lower the threshold for seeking further assistance.

### 3.5 Sustainability

At six-month follow-up, 78% (*n* = 60) of the participants from sets A and B who completed the questionnaire reported that they still frequently applied at least one of the intervention's core strategies to manage their child's fears (refer to Appendix 1 for the participant flowchart with a full overview of the questionnaire response rates). At twelve-month follow-up, this was 70% (*n* = 50). At both follow-up moments, the strategy that most participants still used was the helpful (non-overprotective) approach to cope with the child's inhibited behaviors: 62% and 51% of the participants reported that they continued to frequently use this strategy, respectively. The most frequently mentioned reason for not implementing a strategy anymore was that the child no longer exhibited fearful behaviors or that parents no longer had any personal worries. Other reasons that were mentioned among participants who reported that they did not implement in-vivo exposure through anxiety hierarchies included that they did use this strategy, but in a less structured manner. For example, they did not write down the steps, but incorporated exposure techniques directly into their daily routines. See Table 5 for a complete overview.

**Table 5:** Number of parents who continued to apply the three core strategies of the adapted CLK intervention.

	Six-month follow-up (*n* = 77)	Twelve-month follow-up (*n* = 75)
**How often did parents apply the core strategies of the intervention in their daily lives**	**% (n)**	**% (n)**
Adopting helpful (non-overprotective) approaches to cope with the child's inhibited behaviors		
*Rarely used*	3 (2)	4 (3)
*Sometimes used*	35 (27)	45 (32)
*Frequently used*	62 (48)	51 (36)
Implementing in-vivo exposure through anxiety hierarchies tailored to the child’s fears		
*Rarely used*	40 (31)	48 (34)
*Sometimes used*	40 (31)	38 (27)
*Frequently used*	20 (15)	14 (10)
Practicing ‘realistic thinking’ to address parents’ own worries		
Rarely used	12 (9)	10 (7)
Sometimes used	41 (32)	44 (31)
Frequently used	47 (36)	46 (33)

**Reasons for not applying a strategy (anymore)**	**% (n)**	**% (n)**
Adopting helpful (non-overprotective) approaches to cope with the child’s inhibited behaviors	(*n* = 2)	(*n* = 3)
*Strategy did not work (anymore)*	50 (1)	0 (0)
*There was too little time to implement the strategy*	0 (0)	0 (0)
*Child did not exhibit fearful behaviors (anymore)*	50 (1)	67 (2)
*Parent forgot (how) to implement the strategy*	0 (0)	0 (0)
*Other reason*	0 (0)	33 (1)
Implementing in-vivo exposure through anxiety hierarchies tailored to the child’s fears	(*n* = 31)	(*n* = 34)
*Strategy did not work (anymore)*	26 (8)	15 (5)
*There was too little time to implement the strategy*	16 (5)	18 (6)
*Child did not exhibit fearful behaviors (anymore)*	45 (14)	53 (18)
*Parent forgot (how) to implement the strategy*	19 (6)	15 (5)
*Other reason*	23 (7)	24 (8)
Practicing ‘realistic thinking’ to address parents’ own worries	(*n* = 9)	(*n* = 7)
*Strategy did not work (anymore)*	11 (1)	0 (0)
*There was too little time to implement the strategy*	11 (1)	0 (0)
*Parent did not have any worries (anymore)*	67 (6)	43 (3)
*Parent forgot (how) to implement the strategy*	22 (2)	0 (0)
*Other reason*	0 (0)	0 (0)

*Note.* Participants could select none or multiple reasons for not applying a strategy, so percentages may not sum to 100%.

### 3.6 Context

A major contextual factor that possibly hindered or facilitated the implementation of the adapted CLK intervention was the challenges and opportunities presented by the COVID-19 pandemic, as a large part of the study was conducted during this period. The increased reliance on remote and online solutions during the several lockdowns in this period aligned well with the program's digital format. At the same time, competing demands among parents, such as balancing work and childcare during the pandemic, may have made it more difficult for some to participate. Moreover, (youth) mental health services were under significant strain, which may have affected the promotion and support of preventive programs by professionals as planned.

## 4 Discussion

This study comprehensively assessed the implementation (i.e., recruitment, reach, fidelity, dose delivered and received, satisfaction, sustainability, and context) of an online, targeted, group-based, parent anxiety-prevention program for young children in the Netherlands. The results are promising and provide valuable insights into how the live, online delivery format of the intervention can contribute to the implementation of the intervention in naturalistic circumstances. Nevertheless, several challenges were identified, highlighting opportunities for refinement, improvement, and upscaling in future implementations.

The results showed that the majority of participants were recruited in collaboration with the Public Health Services and child professionals, as would also be the case outside a research context. This indicates that these existing and sustainable recruitment strategies are effective and feasible for referring parents to targeted preventive interventions. Earlier research has similarly reported that community engagement and collaboration with professionals working in mental health, child welfare, or social services contribute to effective recruitment for preventive interventions (Van Doesum et al., 2016; Komanchuk et al., 2023). However, it is important to note that most participating parents in our study were highly educated and of Dutch background, resulting in limited diversity within the population. This aligns with previous research that consistently showed that lower-educated participants and participants with a migration background are often hard to engage and require more intensive, tailored recruitment strategies to ensure their participation in such interventions (Bjørknes et al., 2011; Lingwood et al., 2019; Mian et al., 2015).

A unique aspect of our adapted intervention is its delivery in an online, live group-based format, increasing accessibility while preserving opportunities for connection and interaction among participants. Demonstrating the feasibility of this format is particularly important in the current global context, where challenges such as residing in remote areas, a pandemic or geopolitical conflicts underscore the need for flexible and accessible mental health interventions. By lowering participation barriers, this online approach has the potential to reduce disparities in access to mental health services among diverse populations. This potential is reflected in several findings of the current study. First, attendance rates were substantially higher compared to the earlier translational trial of the original in-person CLK intervention (Bayer et al., 2018), where 34% of participants attended at least five of the total of six sessions, compared with 72% in the current study. This illustrates the accessibility of the adapted intervention. The qualitative interviews reinforced this observation, with participants emphasizing the flexibility and convenience of the online format. Previous studies similarly highlighted the benefits of online interventions in enhancing accessibility (Duppong-Hurley et al., 2016; Flujas-Contreras et al., 2019; Mauricio et al., 2018). Second, parents' active participation and interaction with each other was consistently rated as reasonably to very good (scores between 3 and 4 on a scale of 1–4; see Table 3), suggesting that the format is well-suited in keeping participants engaged. The only exception was the average interaction score for the first session, likely due to its introductory nature. Fidelity and therapists' skills scores were similarly high and stable, indicating a consistent and high-quality delivery of the content as intended. Prior studies on prevention and mental health programs similarly reported high integrity scores with limited variability between therapists (Durlak and DuPre, 2008; Kösters et al., 2017; Chu et al., 2004; Liber et al., 2010). In addition, a meta-analytic review of the ‘FRIENDS’ anxiety prevention program found that the program was more effective when delivered by mental health providers in comparison to non-mental health providers (Fisak et al., 2023), which may also explain the high scores in this study, as all therapists had extensive education and experience in delivering mental health programs. However, drawing definite conclusions based on these findings remains challenging as the observation protocols were specifically designed for the current intervention and integrity research within online preventive programs remains limited. Therefore, future research is needed to confirm these findings and to explore how active participation and fidelity measures can be standardized across interventions.

Other positive findings were the high levels of satisfaction and the fact that the majority of participants reported that they continued to use the intervention strategies in their daily lives to at least some extent well after the program ended. This demonstrates the practical value and sustainability of the skills taught during the sessions. The main reason provided for no longer implementing the strategies was that their child no longer exhibited fears. While this may reflect natural developmental changes in BI over time (Chronis-Tuscano et al., 2009), it may also indicate the potential effectiveness of the intervention. These findings are not entirely consistent with earlier studies on the original in-person format of the CLK intervention, in which 54% of participants reported rarely using the interventions' strategies at one-year follow-up (Bayer et al., 2018). This difference could be explained by the aforementioned higher participation rates in the current study, which might have resulted in a better understanding and application of the core strategies of the intervention.

Several strengths and limitations should be considered when interpreting the results. Strengths included the use of multiple methods and measures that allowed for a comprehensive and reliable process evaluation. Additionally, the hybrid design resulted in a large sample size, increasing the generalizability of the findings. Furthermore, collaboration with relevant stakeholders fostered increased support, facilitating the potential for scaling up the intervention after the study period. A limitation of the study was the underrepresentation of single parent households, parents with a lower educational level, and a migrant background due to possible selection bias. Consequently, the findings are not fully generalizable to the broader population. While the online format enhanced accessibility by resolving logistical barriers during participation (Flujas-Contreras et al., 2019; Mauricio et al., 2018), as reflected in high attendance, engagement, and satisfaction, online delivery alone may not address all aspects of accessibility (Levesque et al., 2013). Specifically, barriers related to the initial intervention entry, including the use of written recruitment materials and the requirement of Dutch language proficiency may have hindered the enrollment among these underrepresented groups (Durlak and DuPre, 2008). Hence, future research should build upon existing recruitment methods by exploring alternative strategies to better reach these populations. Another potential limitation is that response bias, recall bias, and socially desirable answering might have influenced the findings. Participants who were satisfied with the intervention may have been more likely to complete the follow-up questionnaire. To mitigate this, we sent multiple reminders to all parents who initially did not fill in the questionnaire, encouraging them to complete the questionnaires. In addition, we emphasized the importance of their honest feedback. Finally, the qualitative interviews were conducted exclusively with parents from the same intervention group. Although we did not observe any striking discrepancies between the answers emerging from the interviews and the responses to the open-ended questionnaire item administered across all intervention groups, the interview data may not fully reflect the experiences of all participants, as different group dynamics may have influenced their satisfaction.

## 5 Conclusions

This study demonstrated the potential of implementing an online, group-based parenting anxiety-prevention program in a naturalistic setting. The flexibility and accessibility of the online format combined with the group-based delivery were highly valued by participants and contributed to their engagement. These findings underline the promise of online group-based interventions as an effective method for delivering preventive programs, especially in contexts where in-person participation is challenging. The findings also emphasize the importance of tailoring interventions to specific contexts, given that contextual factors can substantially influence both health and implementation outcomes. Even relatively minor adaptations to meet the needs of diverse populations can enhance the suitability and accessibility of an interventions without compromising its core components (Durlak and DuPre, 2008). Therefore, future research should further investigate the potential barriers for single parents, parents with lower educational levels or migrant backgrounds, to determine whether the intervention is equally accessible and beneficial for a broader population. Additionally, investigating how the quality of implementation may affect the effectiveness of the intervention is crucial for refining and optimizing future interventions, and this will therefore be further explored in a follow-up study that builds on the present findings.

## Author contributions

LV, MK, and FS obtained funding for the research project. NK, MK, FS, and LV conceptualized the study and developed the methodological design. NK and LV supervised and coordinated the activities within the study including data collection. NK and MS were involved in gathering the data and conducting the qualitative analyses. NK was responsible for the quantitative data analyses, data validation, visualization and drafting the initial manuscript. All authors thoroughly reviewed and approved the manuscript.

## Funding

This work was supported by ZonMw, the Netherlands Organization for Health Research and Development [grant number 555002021].

## Declaration of competing interest

The authors declare that they have no competing interests.

## Data Availability

The data supporting the current study are stored in DataverseNL and are available upon reasonable request via https://doi.org/10.34894/M8WE6G.
