# Supplementary material for: Early prevention of anxiety disorders in young children: The implementation of a live, online, targeted group-based parenting program
Source: Internet Interv. 2026 May 25;45:100956. doi: 10.1016/j.invent.2026.100956 (PMC13226858; doi:10.1016/j.invent.2026.100956)
Supplement: Supplementary file 1 — Supplementary material [file mmc1.docx]

**Appendix 1. Participant flow and used methods within the Cool Little Kids research project**

**Participant flow and used methods ↓**

**Implementation indicators ↓**

**Recruitment
& reach**

Parents of 201 children fulfilled inclusion criteria

*100%, n = 21*

Three CLK intervention groups were registered in research logs

Ten CLK intervention groups
were registered in research logs

Three CLK intervention groups
were registered in research logs

**Dose
delivered**

*CLK intervention: 99%, n = 77
Control condition: 99%, n = 77*

*100%, n = 24*

*Response rate baseline questionnaire*

**Set A (three groups)**CLK intervention: n = 21

**Set B (ten groups)**CLK intervention: n = 78
Control condition: n = 78

**Actr**

**Set C (three groups)**CLK intervention: n = 24

Parents of 506 children expressed interest in participating in the CLK project, registered in research logs

Between July 2021 and
November 2021

Between June 2023
and May 2024

Participants completed the baseline questionnaire

*76%, n = 16
76%, n = 16*

*CLK intervention: 78%, n = 61
CLK intervention: 71%, n = 55*

*Response rate*

- *Six- and*
- *Twelve-month follow-up questionnaires*

Participants completed the post-intervention questionnaire and
a subset (n = 6) a qualitative interview on their satisfaction
with the adapted CLK intervention

**Satisfaction**

Participants completed the post-intervention questionnaire
on their satisfaction with the adapted CLK intervention

All CLK interventions sessions were observed by the researchers using predefined observation forms. Additionally, parents fulfilled the post-intervention questionnaire with questions about the involvement of their partner outside of session hours

**Dose received & fidelity**

Between December 2021
and May 2023
November 2021

Participants completed a six- and twelve-month follow-up
questionnaire on their long-term use of the intervention strategies

**Sustainability**

*86%, n = 18*

*CLK intervention: 77%, n = 60*

*63%, n = 15*

*Response rate post-intervention questionnaire*

**Appendix 2. Supporting quotes from the qualitative interviews**

1. General satisfaction
   1. *Participant about the therapists of the intervention:*“The therapists guided it well in that regard. They took their time with everyone, also immediately offering tips, tricks or some explanations or assistance. They went through the list with parents very thoroughly. Something I also really appreciated was that they wrote down our children’s names and ages, you could see that they kept a list. That really made you feel their involvement.” P.175
   2. *Participants about the feasibility of combining participating in the intervention with their other responsibilities:*“Every week was a lot, especially for completing the exercises. You don’t have much time. It sounds silly, you have one week to do an exercise but you’re also busy with other things.” P. 178

“I would have preferred to have the sessions all after each other. It did make sense of course because of the Christmas holidays in between. But ideally, I would have preferred to have the training weekly.
‘Interviewer: And what was the reason you would have preferred that?’
Well, because you might be more engaged. I think that if you take a month off, it’s very easy to forget the exercise and not have it as fresh in your mind. So, I think that if you do those repetitive exercises more closely together, it will stick longer. And maybe after a month, you could still do the final session or something. Like, how is it going, and are you still struggling to implement the exercises into your daily life? That is something you could do. But now it was inconvenient that there was always a week in between, and then sometimes not." P.181

1. Content of the intervention
   1. *Participant about the homework assignments:*“I think the learning moments are actually found in the homework assignments. Because then you reflect on your situation to see what’s happening there. I found that to be the part that really connected the sessions to practice” P.182

- 1. *Participant about the activities during the sessions:*"The program provided us with valuable insights and tools. However, I found that the exchange of experiences sometimes took much longer compared to the information that was given." P.162

1. Satisfaction with the environment of the intervention
   1. *Parent about the group-based format*“When I think about school, then [name child] is one of the anxious children. Then I think: am I doing something wrong, you know? After talking to other parents, I think: Oh, it’s nice to know I’m not the only one, that there are other parents are dealing with different things, but also with similar issues. So, I really appreciated that you can learn from each other. At least that is how I felt, freer to share things. Another parent who doesn’t have those problems might think: can’t he really do that? And I think: No, he is just really scared to do a lot of things." P. 179
   2. *Parents about the online format:*“With an online meeting, it works well in terms of time management. I needed to tidy up the counter and load the dishwasher, so I would say: Hey sorry, I am a bit behind, so I’ll just listen to you. My wife has also listened a few times, just while ironing. […] it saved me travel time to [city name]. It was a bit out of the way, and if I had to leave half an hour or maybe 45 minutes earlier, and if there is a lot of traffic you really need to leave on time, or you can’t attend. So, I think the online sessions had more benefits than downsides.” P.175

      "I think live sessions could be fun too, doing it all together in one room. But I also understand that this is much easier. So, I didn't find it disruptive, especially since we’ve all been doing it online for two years during COVID. The initial novelty has worn off, but I would also enjoy having a live session once.
      ‘Interviewer: And if the sessions were live during the day, would that be something you would attend?’
      Yeah, well, that’s the tricky part. Everyone has to work, so when would you do that? You might need to arrange childcare, some people might be single parents, so yeah, that could be an obstacle." P.178
2. Satisfaction with the self-assessed attainment of the goals of the intervention
   1. *Parent about the perceived changes in her child*“I do feel that she is making progress because of this. And there is more understanding now, both from me and from her father, to whom I also explained what we should do how it should be done.” P.182
